# Survivorship dynamics of the flora of Devonian Angarida

**DOI:** 10.1098/rspb.2022.1079

**Published:** 2023-01-11

**Authors:** Elizabeth M. Dowding, Nikolay Ivanovich Akulov, Irina Mikhailovna Mashchuk

**Affiliations:** ^1^ Centre for Earth Evolution and Dynamics, University of Oslo, Blindern, 0316, Norway; ^2^ GeoZentrum Nordbayern, Department of Geography and Geosciences, Friedrich-Alexander Universität Erlangen-Nürnberg, Erlangen, Bavaria, Germany; ^3^ Siberian Branch of the Russian Academy of Sciences, Institute of the Earth's Crust, 664033 Irkutsk, Russia Federation

**Keywords:** phytogeography, Devonian, Siberia, Large Igneous Province

## Abstract

Devonian plants in Siberia present protracted pioneer succession. Research into the survivorship dynamics of early plant communities upon the palaeocontinent Angarida have demonstrated that transgression and volcanogenic nutrient influx were key to the survival of colonizing plants. Taxic proportions show that migrating taxa entered Angarida from the southwest, Kuznetsk and Minusinsk basins, dispersing across the continent in waves through central areas northwards. The patterns of dispersal are consistent throughout the Devonian. Increased nutrient load from the active pulses of the Viluy-Yakutsk Large Igneous Province, biogeomorphic ecosystem engineering and the increased biomass of Angaridan plants are assisted by Late Devonian transgression. These cumulative factors can be linked to the Late Devonian marine extinctions observed in Siberia.

## 1. Introduction

Survivors of extinctions and biotic events are at once remnants and progenitors. During the Devonian (419–358 Ma), land plants were establishing their dominance and ‘terraforming’ terrestrial environments [1]. Involved in the process was an increase in diversity and complexity in floristic communities, a rise in biomass, and an associated suite of abiotic repercussions [2–4]. In modern terrestrial ecosystems, the colonization of new environments by floral communities can facilitate a shift from barren landscape to multi-layered complex systems [5,6]. The Devonian is a unique period in that this process is protracted, the forests and multi-storey terrestrial systems established during this period were the first in Earth's history and relied on origination in addition to migration. Within this study, we determine the phytogeographic survivorship dynamics of Siberia through this key period.

**Figure 1.**
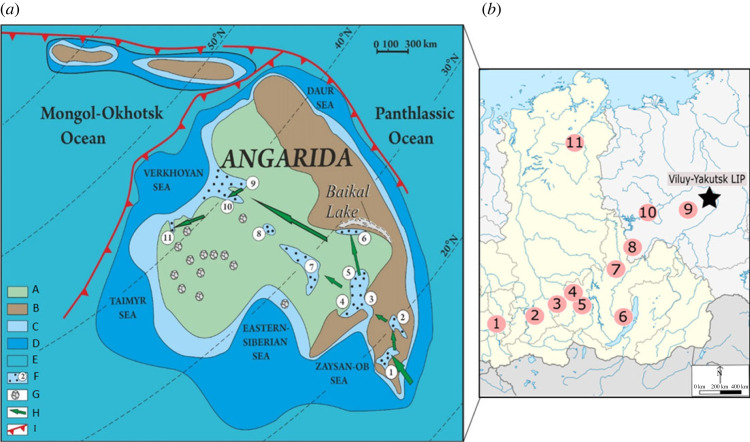
(*a*) Map of Devonian Siberia modified from Akulov & Mashchuck [7]. Sedimentary basins 1–11: 1, Kuznetsk; 2, Minusinsk; 3, Rybinsk; 4, Kansk-Taseevsk; 5, Poimo-Biryusinsk; 6, Pribaikalsk; 7, Angara-Tungussk; 8, Ichodinsk; 9, Kempendyaysk; 10, VIgyattinsk; 11, Kyutyungdinsk; A–B, land: A, lowlands and plains; B, mountains; C–D, sea: C, shallow; D, shelf; E, deep; F, sedimentary basins; G, kimberlite volcanism and diamond-bearing fields; H, direction of the trajectory of colonization of the continent by higher plants; I—subduction zones. (*b*) Modern Siberia with sedimentary basin location, numbering identical to figure 1*a*. (Online version in colour.)

Throughout the Devonian, Siberia (figure 1*a*) was relatively isolated with few endemic taxa [7]. Located in the tropical palaeoclimatic belt, the Siberian continent moved in a submeridional direction with clockwise rotation at 30° N [8–14]. A hot semi-arid climate dominated its territory. Tectonic-magmatic activation that began in the Late Silurian-Early Devonian on the Siberian continent, caused uplift, rainshadows and the formation of the vast land area of Angarida [15].

On Angarida, erosion and denudation processes dominated, facilitated by transgression. Facies analysis of Devonian deposits exposed in sedimentary basins testifies to the redeposition of various horizons of Lower Palaeozoic deposits in lacustrine conditions [15,16]. The first terrestrial vegetation grew in the coastal parts of the Devonian sedimentary basins of Angarida. The change of transgressive and regressive processes continued throughout the entire Devonian period until the beginning of the Pennsylvanian epoch, which was reflected in the accumulation of sediments in sedimentation basins [17,18]. The largest of them, Rybinsk, gradually became shallow, and in the Mississippian era ceased to function [16]. These events greatly complicated the development of higher vegetation, but nevertheless, it appeared.

Devonian and Early Carboniferous continental deposits were formed within 11 palaeobasins within Angarida (Angaraland) which was an extensive 4 million km landmass [7,12,16]. Although not all the basins were active during the Devonian and Early Carboniferous, the stratigraphic record shows a consistent pattern of volcanic activity in the palaeo-northeast of Angarida, periods of basin flooding and desiccation, and the Middle Devonian colonization of continental Siberia by higher plants [7,16]. Survivorship and phytogeography within Siberian systems is consequently influenced by the interactions of migration with environmental stressors. Those taxa which survived and were able to migrate would then give way to the Carboniferous Angara flora (e.g. *Angaraopteridium* and *Chacassopteris*) of the Boreal realm [19].

In the Early Devonian, the primary taxa were Zosterophyllopsids and *Psilophyton*, living in alluvial freshwater conditions [20–22]. While the distribution of taxa was relatively high for the Early Devonian (on par with Norwegian systems; [23]), a rapid transgression during the early Emsian flooded the landmass and diminished regional vegetation cover [24–26]. Alterations between transgressive and regressive cycles continued throughout the Devonian and into the Carboniferous. These changes in habitat availability on Angarida were compounded by tectonic action (rifting and passive margins in the east and southwest) and the Late Devonian active pulses of the Viluy-Yakutsk Large Igneous Province (LIP) [27].

In this study, we aim to assess the Frasnian-Tournaisian biogeographic relationships of Angarida, discuss floristic survival and migration dynamics, and provide context for their corollary effects on the marine realms (Late Devonian biocrises) and the key Carboniferous Angara flora.

## 2. Methods

The occurrences of over 280 species were collected across the Siberian platform (figure 1*b*) between 419 and 358 Ma. These data were then treated at species-level Survival Analysis using the Kaplan–Meier Estimate (KMSA). To highlight Frasnian-Tournaisian changes, the data were analysed by running a stepwise KMSA for every time-bin, e.g. Early Devonian–Middle Devonian, Early Devonian–Frasnian, etc. until Tournaisian which was then repeated with stepwise binning (e.g.‘Middle Devonian’) to create 11 survival probabilities using the following formula:

$$S_{t+1}=S_{t}\frac{N_{t+1}-D_{t+1}}{N_{t+1}},$$

where *S_t_* is the probability of survival; *N_t_* is the number of threatened/still extant taxa; *D_t_* is the number of taxa that become extinct in the region during the next interval.

**Figure 2.**
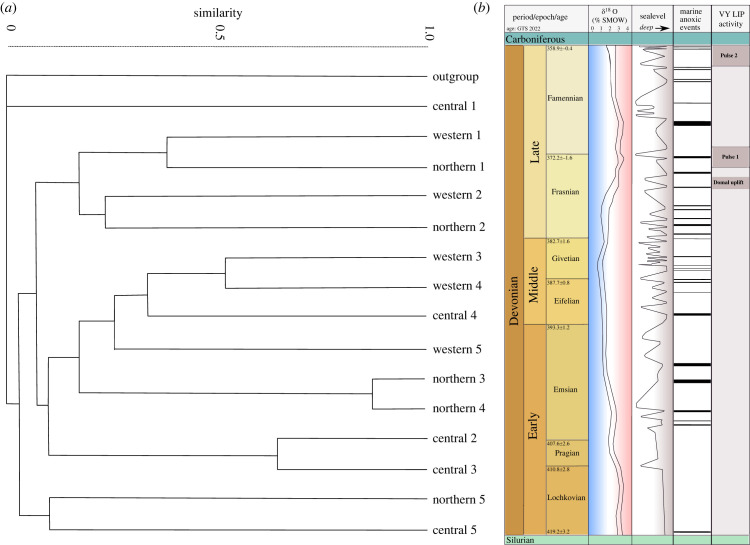
(*a*) Cluster analysis of Siberian temporal areas from the Early Devonian to Tournaisian; where central, northern and western relate to basin grouping and, ‘1’ is Early Devonian, ‘2’ is Middle Devonian, ‘3’ is Frasnian, ‘4’ is Famennian and ‘5’ is Tournaisian. Confidence index: 0.8741. (*b*) Integrated summary modified from [28]. From left to right, stage/age boundaries [29], conodont apatite oxygen isotopic composition [30], carbon isotope composition [29], relative sea level [29] and Viluy-Yakutsk LIP activity [31]. (Online version in colour.)

Within this analysis, absence across two temporal bins was assumed as ‘regional extinction’ and taxon absence across only one time-bin was counted as ‘sampling error’ and the taxon was recorded as present. The Late Devonian was considered both at epoch and stage level. The KMSA proportions are then compared to the number of ‘first appearances’ which is a cumulation of the number of taxa that first become present between the time-bins. The temporal difference between the Frasnian, Famennian, and Tournaisian is a maximum of 4 million years. While this is not an insignificant amount of time, the purpose of the KMSA is to test for survivorship between treatments. In the case of the flora of Angarida, the treatment is the LIP activity (Pulses 1 and 2, figure 2*b*) and marine extinction, which are synchronous with the chronostratigraphic chart's temporal bins.

After temporal binning, the data were then divided into directions according to the modern orientation of the platform, where ‘north’ represents Kempendyaysky, Ygyattinsky, Kyutyungdinsky districts, ‘central’ - Kansko-Taseevsky, Poimo-Biryusinsky, Pribaikalsky, Angara-Tungussky, Ichodinsky districts and ‘west’ Kuznetsk, Minusinsk and Rybinsk depressions [16]. The phases of these units are detailed within the following section. The division of Angarida into directional and temporal subsets within this paper allows neighbouring fossiliferous deposits to fill gaps caused by depositional inconsistencies. Proportions of taxa shared between the north, central and west units were calculated, then these data were grouped according to TAAp [32] and analysed using cluster analysis (unweighted pairwise group mean, Sørenson index). Metrics were chosen to establish ‘presence-based’ relationships and lessen absence signification.

## 3. Results and Discussion

### (a) Interrelationships and dispersal histories

The affinities of the basins of Angarida significantly alter through time. Transitions in flora type in Siberia is generally accepted as *Psilophyton* (Lower Devonian), *Hyenia* (or *Protopteris*; Middle Devonian), *Archaeopteris* (Late Devonian) *Sublepidodendron* and *Lepidodendron* (Tournaisian), with diversity highest in the western, coastal zones [21]. Previous studies [26,33,34] have proposed that these floristic transitions began with dispersal and migration from the southwest basins (Kuznetsk and Minusinsk) through the centre of Angarida, heading northward (figure 1*a*). The proportions of survivorship across Angarida (table 1) show substantive shifts in species composition and support the influence of southwest-north migration across the Siberian platform.

**Table 1.** Kaplan–Meier Estimate survival proportions.

time-bin (Devonian)	species per time-bin	survivor count per time-bin	survival proportions % (KMSA X 100)
Early – Middle	37	17	45.95
Early – Frasnian	—	7	18.92
Early – Famennian	—	6	16.22
Early – Tournaisian	—	0	0
Middle – Frasnian	90	31	34.44
Middle – Famennian	—	21	23.33
Middle – Tournaisian	—	7	07.78
Frasnian – Famennian	47	33	70.21
Frasnian – Tournaisian	—	8	17.02
Famennian – Tournaisian	58	22	37.93
Late – Tournaisian	77	33	42.86

Higher plants first occur in Laurentia during the Pridoli, penetrating the Angarida landmass from the west through Kuzbass (Lochkovian) and then the Minusinsk basin (Middle Devonian [7] table 2; figure 2*a*). Competitive replacement of flora through migration and radiation have often been cited as an influential factor in floral systems and Devonian phytography, e.g. the replacement of *Zosterophyllopsida* by lycopsids and basal euphyllophytes in the late Pragian of Siberia [22]. Taxa present within the northern basins are shared at higher proportions with the west (table 2) than those of the central basins (table 2). The western and central basins were the richest in terms of species diversity (162 and 110 species, respectively). The northern basins, with markedly fewer occurrences and less richness, is shown to have sourced its diversity from the western basins (81.4%). Consequently, the northern and western basins form the apical grouping of the clade at both Early and Middle intervals (figure 2*a*).

**Table 2.** Count of shared species and proportion of shared species by unit.

		areas		
		north	west	central
area combination	species shared	43	162	110
north + west	35	81.4%	21.61%	-
north + west + central	11	25.6%%	6.79%	10%
west + central	33	-	20.37%	30%
north + central	12	27.91%	-	10.91%

The distribution of taxa between the three units (west, central and east) and 11 basins shows that very few taxa were shared across all Angarida (table 2). This is associated with isolation and the difficulty in floral migration caused by the Yenisei, Sayan and Baikal Mountains, which prevented penetration into the continent. However, if during periods of transgression the taxa were able to disperse, the drying reservoirs provided organic silty substrates which acted as a natural fertilizer for the growth of the terrestrial plants on Angarida [16]. Migration of higher plants and competitive taxa into Angarida suggest that the central basins were resistive to colonization by migration from the west, compared to northern basins, or the migration into central regions was more difficult owing to the aforementioned abiotic features. The Devonian patterns of floristic colonization of Angarida are potentially linked to patterns observed in the Carboniferous Angara flora.

In considering the biogeographic evolution of the Devonian-Carboniferous system, it should be noted that studies into the preservation potential of transgressive sequences have shown a positive relationship between transgression and taxic richness and diversity and the inverse for regression [35–37]. Within this study fossil flora during the Frasnian-Tournaisian was consistently observed within the basins of the southwest and more rarely upon the Angarida continent [16]. Preservation biases are therefore most likely to appear in northern basins [16,38]. However, the patterns observed within the paper, linking transgression with increased range can be disentangled from preservation bias by the biogeographic history of the plant arrivals. Though deposition is consistent within the northern basins [16], the arrival of *Archaeopteris* and *Archaeocalamites* are exclusively from the southwestern basins, from where they colonized Angarida. Throughout the Devonian there were no endemic Siberian flora, and so tracking their passage across Angarida can be achieved in conjunction with linage histories.

The origination of endemic Angaridan flora did not begin until the Carboniferous (Visean; [7,39,40], among others). It is from the survivors of the Devonian that the globally significant Angara flora, e.g. *Angaraopteridium* and *Chacassopteris*, would develop. Additionally, summaries of the macrofloral zones of the Angaran realm show that the best representations of the floral complexes are to be found in the southwestern basins, Minusinsk and Kuznetsk [39,41], which show diversity patterns remain consistent throughout the Carboniferous. In the upper Visean-upper Serpukhovian, the Angaraopteridium zone can be identified in central basins [40]. The consistency of the pattern throughout the Devonian and Carboniferous (migration from western to northern basins) suggests abiotic limitations to dispersal and facilitation of range expansion through transgression.

### (b) Angarida and Late Devonian extinction

The Late Devonian of Angarida was extremely turbulent with transgressive phases reducing the amount of habitable land and instability owing to effusive volcanics related to LIP activity and the Eastern passive margin [17,42,43]. Consideration of the temporal constraints within survivorship (table 1; figure 2*a*) suggests that the western basins may have acted as a refuge during the transgressive phases and active pulses of the Late Devonian Viluy-Yakutsk LIP. Figure 2*a* shows that the Tournaisian west area is nested within a Devonian clade suggesting that a higher proportion of Devonian taxa survived within the western basins into the Carboniferous than in the central and northern units. However, this interpretation should be compared to the observation of the rapid proliferation of higher plants throughout the Carboniferous of Siberia.

During the Early Devonian higher plants are first recorded in Siberia and throughout the Middle Devonian they proliferated and formed a significant part of the ground cover [7,21,24]. The Early Devonian taxa were less able to survive and were subsequently replaced in the turbulent conditions of the Middle Devonian to Tournaisian than the higher plant colonizers (table 1). Early and Middle Devonian taxa had the lowest survival probabilities recorded into the Carboniferous (0% and 7%, respectively). While this in part is owing to the rarity of long-lived taxa, the low retention of genera across the Middle Devonian–Frasnian boundary coupled with high richness in the Frasnian suggests that the turnover is related to the colonization of Angarida by higher plants [26]. The timing of this is in keeping with the migration of higher plants, such as *Archaeopteris* and *Archaeocalamites,* onto Angarida during the Middle Devonian [44,45]. Throughout the Middle and Late Devonian, these higher plants spread from west to north establishing Angarida's emergent complex and multi-storey plant communities [44,45]. Comparison of interval bins through the KMSA (e.g. table 1) to show baseline turnover points toward changes in survivorship owing to ecological succession.

Results indicate that after the loss of Middle Devonian taxa (approximately 66%) and during environmental perturbations including transgressive-regressive (T-R) cycles and volcanic activity, richness in the Late Devonian increased through migration (table 1). The strong relationship of the Late Devonian units to each other ((northern 3, northern 4) and (western 3, western 4); figure 2*a*) show spatial and temporal separation of the basins. Between the Frasnian and Famennian intervals, 70% of Angaridan flora survived (table 1). This was then followed by a marked decline into the Tournaisian with only 17% of Frasnian taxa crossing into the Carboniferous. New arrivals during the Famennian, however, had a much higher success in crossing into the Carboniferous (37%, table 1). It may be that the western areas, rather than purely being refugia from extinction, represent a transitional region between waves of colonizing plants and their expansion into central and northern Areas.

This view is supported by the changes in species richness and survivorship during coeval transgressive phases and LIP active pulses. Throughout the Late Devonian, transgression expanded the basins of Angarida increasing the distribution of plants across the continent. Ershova *et al*. [18] have shown that during this key period, evaporitic and lagoonal areas transitioned into shallow sea in northern areas. These periods are also associated with the emplacements of tuff, felsic volcanics and granitoids [46]. Though these events were initially destructive, survivorship within the Late Devonian was consistently high for single-bin intervals, the Frasnian-Famennian interval retaining 70% of its species (table 1). Examples from the Columbia River Basalt Province (CRBP), a Miocene LIP in the USA, shows that volcanogenic eutrophication is contemporary (10^3^ year duration interbeds) with eruptive activity within the LIP [47]. Additionally, nutrient deficiency and decreased richness was identified in the interbeds of the CRBP under conditions similar to Angarida [47].

The increased survivorship during the Frasnian-Famennian interval shows that, contrary to being detrimental to the pioneer botanical systems, transgression and LIP activity may have facilitated migration and establishment.

### (c) Influence of floral survival dynamics on global extinction

Discussion of phytographic survival dynamics within the Devonian is complicated by a lack of underlying baselines. The rates of background extinction, turnover and origination have not yet been established for Angarida during this period. This is owing, in part, to the unique nature of the nascent Devonian floral systems. The colonization of land by higher plants was in its early stages and ecological feedbacks, niche development and establishment of multi-story systems were processes secondary to ‘terraforming’ or ‘biogeomorphic ecosystem engineering’ [48,49]. In modern ecology, there are first- and second-order pioneer communities (see [50] for early theory and [51] for modern palaeontological example [51,52]). Within the Devonian the establishment of complex terrestrial ecosystems caused this ‘pioneer’ process to be drawn out. The survivorship and distribution of higher plant communities across Siberia brought about changes in the spread of soils and silicate weathering [53,54], river morphology [55], and the increase of terrigenous-to-aquatic sources of organic matter, vitrinite and inertinite [56] among others. The arrival of higher plants acted as a delayed progression to ‘intermediate’ community establishment and changed the impacts of terrestrial environments on the marine.

The Late Devonian biocrises, the Kellwasser and Hangenberg events, primarily effected shallow marine ecosystems [57,58]. In the Late Devonian of Siberia, Kellwasser and Hangenberg facies are observed through the deposition of black mudstone and black limestones, and black shales, respectively [14,16,18,59]. Associated with these layers in various locations (and combinations) are bituminous deposits, effusive tuff, black siltstones, shales and mudstone [46]. Though regional T-R cycles differ [60], evaporites and coal were both were well distributed across Siberia during the Late Devonian and Tournaisian ([16,18,27,61]; among others). Periods of recovery, migration and increased survivorship are associated with increased available habitat (table 1; see (Frasnian-Famennian; FF) and (Famennian-Tournaisian) proportions). The success of the terrestrial flora led to increased density and distribution of terrestrial biomass across Angarida. This success would result in the release of nutrients into shallow marine systems. Volcanogenic and biogenic eutrophication are known influencing factors of the FF extinction [48,56,57].

The cause of FF boundary extinction is contentious (see [62] for comment). Within Laurentian samples, peaks in nutrient input associated with land plants were correlated to the Upper Frasnian Kellwasser events (see [57]). The Laurentian conditions were similar to Siberia, owing to shared generic profiles and Siberia's lack of endemic species [7]. In both the Laurentian and Siberian successions, *Archaeopteris* was the dominant arborescent lignophyte, expanding its distribution and diversity throughout the Late Devonian. Recent work by Lu *et al*. [57] has shown that the terrestrial expansion of floral communities, resulting in augmented terrestrial-to-marine fluxes and recurrent anoxic conditions related to the Upper Kellwasser biocrises in Laurentian areas. Within Siberia, the arrival of higher plants, increase in species diversity and periods of increased survivorship (table 1; (FF)(Famennian-Tournaisian)) show similar patterns and trajectories to the Laurentian examples. While the authors believe that marine extinctions within the Siberian successions were impacted also by transgression and the active pulses of the Viluy-Yakutsk LIP, floral survivorship and community dynamics are believed to be a significant factor in the biocrises. Associated with this view is the recommendation for future study of the influence of early establishment phytogeography, the ranking of Devonian pioneer systems, and the influence of floristic density and distribution on the marine realm.

## 4. Conclusion

Angarida during the Devonian was an unstable environment for migratory plants species. Through the Devonian the dispersal was facilitated across Angarida by transgressive phases and the expansion of floristic communities by regression. This process brought higher plants through the Kuznetsk and Minusinsk basins from Laurentia and Kazakhstan through the Zaysan-Ob Sea, moving through the central basins to the northern near the Verykhoyan Sea. Comparison of shared taxa between the basins shows that the northern basins draw their diversity from the west; the separation of western and central basins recording waves of floral colonization over Angarida.

Survivorship across Angarida was favoured in the western areas. The increased survivorship in these areas was not because of a refugia offered by the environment but rather owing to the larger proportion of higher plants originating outside of Angarida within these areas. Significant loss of species richness across Angarida is not tied to active phases of the Viluy-Yakusk LIP. Instead, the active phases of the Viluy-Yakusk LIP increased survivorship at the FF boundary to its highest levels through the influx of volcanogenic nutrients into the Devonian basins. The nutrient influxes associated with the two active pulses were synchronous with a transgression, permitting both colonization across Angarida and sufficient nutrient load for establishment. It is likely that these conditions were more important in Devonian phytogeographic systems than future systems owing to the comparatively low taxonomic and morphological diversity of early plants in addition to Angarida's reliance on migration not origination. The favourable conditions, increased richness and high survivorship in the Late Devonian facilitated a proliferation of plants, increasing biomass and altering the spread of soils and silicate weathering, river morphology and increased terrigenous-to-aquatic sources of organic matter. The increased nutrient load, both from volcanogenic and terrestrial biogenic sources, is a likely factor in the marine extinctions observed in Siberia during the Late Devonian [48,56,57].

Angarida presents an excellent opportunity to study the interplay between biogeographic transition, LIPs, and early plant systems resilience to environmental perturbations. Future study includes geobiological analysis of the Devonian sediments during the Late Devonian to better address the correlations between LIPs, transgression, and the survivorship of plants.

## Data Availability

The data are available from the Dryad Digital Repository: https://doi.org/10.5061/dryad.rfj6q57fc [63]. The data are provided in the electronic supplementary material [64].
